# Risk of Infections With Long-Term Left Ventricular Assist Device Support

**DOI:** 10.7759/cureus.41412

**Published:** 2023-07-05

**Authors:** Rajendra Karnatak, Uriel Sandkovsky

**Affiliations:** 1 Infectious Disease and Critical Care Medicine, Aurora Health, Milwaukee, USA; 2 Division of Infectious Disease, Baylor University Medical Center, Dallas, USA

**Keywords:** risk factors for infections post lvad implantation, infections with long term lvad support, vad related infections, vad specific infections, lvad infections

## Abstract

The average life expectancy post-left ventricular assist device (LVAD) implantation has significantly increased in recent years. Impaired cellular immunity post-LVAD implantation has been suggested. It is not clear if a prolonged duration of LVAD support will lead to an increase in infections and possibly cause opportunistic infections, as seen in immunocompromised patients.

Methods: We retrospectively reviewed all the patients who underwent new continuous-flow (C-F) LVAD implantation between January 1, 2013, and December 31, 2014, at the University of Nebraska Medical Center. Patients were followed until heart transplant, LVAD explantation, death, or December 31, 2017. We defined LVAD infections as per the International Society of Heart and Lung Transplantation (ISHLT) definition: VAD-specific, VAD-related, and non-VAD infections. The primary outcome was to calculate the incidence of LVAD infections per 1000 days of LVAD support. Secondary outcomes were to assess the cause of death and the effect of bloodstream infections on LVAD thrombosis, stroke, and death.

Results: During the study period, a total of 94 patients underwent a C-F LVAD implantation. Five patients were lost in follow-up; 89 patients were included in the study. The mean age at LVAD implantation was 54 (SD+15) years. Out of 89 patients, 67 (75%) were men, and 53/89 (71%) received LVAD as destination therapy (DT). At the time of LVAD implantation, 34/89 (38%) patients had ITERMACS (interagency registry for mechanically assisted circulatory support) score 1 (cardiogenic shock). The median duration of LVAD support was 387+493 days, with an interquartile range of 140 to 1083 days. The incidence rate of infections post-LVAD implantation decreased from 3.2 /1000 LVAD days (95% confidence interval [CI] 2.54-4.03) during the first year of LVAD support to 0.78/1000 LVAD days (95% CI, 0.38-1.65) during the following third year of LVAD support. Similarly, the incidence of VAD-specific infections in the first year post-LVAD implantation versus the third-year post LVAD implantation decreased from 0.83/1000 LVAD days (95% CI, 0.53-1.30) to 0.33/1000 LVAD days (95% CI, 0.10-1.04). On univariate survival analysis, an increased risk of death was associated with a one-year increase in age at LVAD implantation (hazard ratio (HR) 1.05 (95% CI, 1.01-1.09), p=0.01), the presence of infection within 30 days before LVAD implantation (HR 2.44 (95% CI, 1.09-5.48), p=0.03), underlying ischemic cardiomyopathy (HR 2.96 (95% CI, 1.28-6.80), p=0.01), and lower ITERMACS profile HR 3.64 (95% CI, 1.09-12.13, p=0.04). Bloodstream infections (BSIs) were not associated with an increased risk of death (HR 1.63 (95% CI, 0.56-4.80, p=0.37). Univariate survival analysis for poor outcomes (LVAD thrombosis, stroke, or death) showed BSIs increased the risk of having a poor outcome (HR 2.39 (95% CI, 1.02-5.57), p=0.04).

Conclusions: The incidence rate of post-LVAD infections decreased significantly over time. LVAD implantation may not be contributing to immune suppression as previously suggested. In our study, BSIs were found to have a significantly increased hazard ratio for a poor outcome post-LVAD implantation.

## Introduction

This article was previously presented as a meeting abstract at the American Thoracic Society (ATS) Annual Scientific Meeting on May 21, 2019, in Dallas, Texas. Heart failure is a major cause of morbidity and mortality in the United States. As per the American Heart Association’s 2016 report, about 5.7 million people are living with heart failure in the United States, and worrisome projections indicate up to 8 million people will have heart failure in the US by the year 2030 [1]. Worldwide, it is estimated that 26 million people are living with heart failure, and this number is underestimated as data from low- to middle-income countries is not reliably available [2]. The 2011 Centers for Disease Control and Prevention hospital discharge survey (2000-2010) indicated an increasing number of young patients (age<65) are now being diagnosed with heart failure [3]. Up to 50% of patients will die within five years of a heart failure diagnosis.

Heart transplantation is the definitive therapy for the treatment of end-stage cardiomyopathy. Due to the huge organ shortage, most patients die waiting for an organ transplant. Alternatively, mechanical circulatory support (MCS) is being used to treat advanced heart failure patients. The left ventricular assist device (LVAD) is the most common type of MCS used. Various strategies to use LVAD have been designed, such as bridge to transplantation (BTT), destination therapy (DT), or bridge to decision. In recent years, a significant increase in LVAD implantation has been noted [4]. In 2005, the Interagency Registry for Mechanical Assisted Circulatory Support (INTERMACS) was established to acquire data on clinical outcomes in patients receiving an FDA-approved MCS for the treatment of advanced heart failure [5]. In 2011, the International Society of Heart and Lung Transplant (ISHLT) defined MCS infections into three major categories: Ventricular assist device (VAD)-specific infections are defined as infections that are specific to VAD patients and do not occur in non-VAD patients (e.g., pump, cannula, pocket, or drive line infections); VAD-related infections are defined as infections that can occur in patients without a VAD but need special consideration with respect to the presence of a VAD (e.g., mediastinitis, bloodstream infections, and infective endocarditis); and non-VAD are infections defined as infections that are not affected by the presence of a VAD, e.g., UTI, pneumonia [6]. Although long-term infectious complications related to LVAD implantation are not well studied.

Over the last decade, the type of LVAD support used and survival post-LVAD implantation have changed drastically. With the introduction of newer continuous flow (CF) LVAD implantation, one-year survival has reached up to 83%, and five-year survival has reached up to 50% [4]. The most recent INTERMACS analysis indicated that CF LVAD implantation as a destination therapy (DT) indication has increased from 46% to 73% from 2012 to 2017 [4]. Earlier reports have suggested LVAD-associated impairment of cellular immunity [7]. Very little data is available to assess the effect of prolonged LVAD support on the risk of infection. An INTERMACS report mentioned that 8% of deaths were considered a consequence of a major infection (sixth most common cause) in patients receiving a continuous flow (CF) LVAD during the years 2012 to 2017 [1]. Moreover, the INTERMACS report also mentioned that infection-related deaths are most likely underestimated, and it is not clear if infections coincide with other common causes of death, such as stroke, multi-organ failure, or withdrawal of support [1].

We conducted a study aimed at evaluating the incidence of infections post-CF LVAD implantation and assessing risks of infections with prolonged CF LVAD support. We also aimed to evaluate if bloodstream infections coincided with the most common complications post-LVAD implantation, such as LVAD thrombosis, stroke, or death.

## Materials and methods

The study was approved by the University of Nebraska Medical Center’s (UNMC) institutional review board. We retrospectively reviewed all patients who underwent a new CF LVAD implantation between January 1, 2013, and December 31, 2014, at our institution. Patients were followed until heart transplant, LVAD explantation, death, or December 31, 2017. We defined LVAD infections as per the ISHLT definition: VAD-specific, VAD-related, and non-VAD infections. For each patient, information regarding demographics (e.g., age, race, and gender) and clinical characteristics were obtained, and if applicable, type of LVAD infection, date of LVAD infection, type of LVAD complication, date of LVAD complication, date of transplantation, date of death. Table 1 depicts baseline characteristics for patients in our cohort.

**Table 1:** Baseline Characteristics of Patients Underwent Continuous Flow LVAD Implantation * INTERMACS definitions: I: Cardiogenic sock, II: Progressive decline on ionotropic support, III: Stable but inotrope dependent, IV: Resting symptoms, V: Exertional intolerance, VI: Exertional limitation, VII: New York Heart Association class. BMI: body mass index; BTT-bridge to transplant; CKD-chronic kidney disease; DT- destination therapy; LVAD- left ventricular assist device.

Characteristic (%)	Number
Age, median (range)	54 (17-76)
Male	67 (75)
Race	
Caucasian	78 (88)
African American	5 (5)
Other	6 (7)
BMI, median (range)	31 (18-52)
Underlying Cardiomyopathy	
Ischemic	41 (46)
Non-Ischemic	38 (44)
LVAD Placement Plan	
Emergency	27 (30)
Elective	52 (70)
Goal of LVAD Placement	
BTT	26 (29)
DT	53 (71)
INTERMACS profile	
1-3	50 (56)
4+	39 (44)
Comorbidities	
DM	29 (33)
CKD	19 (21)
Morbid obesity	10 (11)
Need for RRT post op	11 (12)
Operative time, median (range)	238 (122-633)
Survival at 36 months	22 (25)
Heart transplantation	45 (51)
Total LVAD days, median (range)	50483,387 (2-1593)

The primary outcome was the incidence of LVAD infections (VAD-specific, related, and non-VAD) per 1000 days of LVAD support, which was calculated each year for three years of post-LVAD follow-up. Secondary outcomes were to assess risk factors for bloodstream infections (BSIs) and if BSIs served as a risk factor for common LVAD complications, including death or poor outcomes (including stroke, thrombosis, or death). Each episode of infection was counted only once; for example, if a bloodstream infection was from an LVAD driveline infection, it was only counted once in the overall analysis. Similarly, if the same pathogen was involved in repeated bloodstream infections while LVAD was in place, each episode was counted only once (a repeat episode was considered a relapse).

Inclusion criteria: All patients who underwent continuous flow LVAD implantation at the UNMC from Jan 1, 2013, to Dec 31, 2014, were included in the study.

Exclusion criteria: Patients with pulsatile flow LVADs and patients with LVAD replacement were excluded from the study. Patients were excluded if post-LVAD implantation follow-up was not done at UNMC. Patients with a total artificial heart were also excluded from the study. 

Statistical analysis: Incidence rates were calculated for three distinct year-long periods: the first, second, and third years of follow-up post-LVAD. To calculate incidence rates, patient days (the denominator) were defined by the total number of days a patient was followed up within a given time period (for example, if a patient was followed for 1 year and 14 days, they would contribute 365.25 patient days for the incidence in year one and 14 patient days for the incidence in year two). The number of infections was counted within a given period, and one person may have contributed multiple infections to the numerator within a given period. The numerator was divided by the denominator and then multiplied by 1000 to be able to generate the rate of infection per 1000 LVAD-patient days for a specific period of follow-up. For the secondary analysis, Cox Proportion Hazards regressions predicting time from LVAD implantation to BSI, death, or poor outcome (i.e., stroke, thrombosis, or death) were run with a single predictor at a time; due to the low occurrence of outcomes, multivariable modeling was not feasible, so results are presented as unadjusted hazard ratios with 95% confidence intervals. The BSI models were run as competing risk models, where death was treated as a competing risk. For the other two models, BSI was treated as a time-varying covariate since its status was unknown at the start of the follow-up. All analyses were performed using SAS software version 9.4 (SAS Institute Inc., Cary, NC).

## Results

During the study period, a total of 94 patients underwent C-F LVAD implantation. Five patients were lost in follow-up, and 89 patients were included in the study. The mean age at LVAD implantation was 54 (SD+15) years. Sixty-seven of 89 (75%) were men. 53/89 (71%) received LVAD as DT. 34/89 (38%) patients' INTERMACS score was 1 (cardiogenic shock) at the time of LVAD implantation. During the study period, a total of 50483 days of LVAD support were provided. The median duration of LVAD support (+SD) was 387+493 days, with an interquartile range of 140 to 1083 days.

There were 97 episodes of total infections (VAD-specific, VAD-related, or non-VAD) identified in 51 (57%) patients. Out of the 97 episodes of infections, 23 (23.7%) were VAD-specific, 16 (16.4%) were VAD-related, and 56 (57.7%) were non-VAD infections.

**Figure 1:**
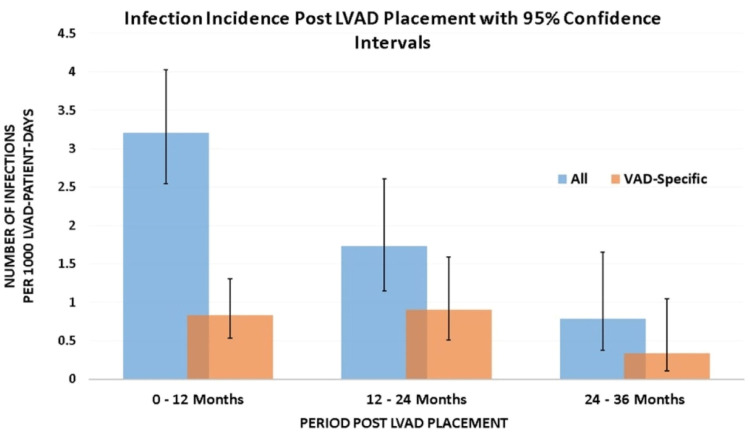
Incidence rate of LVAD infections over time

The incidence rate of infections post-LVAD implantation decreased from 3.2/1000 LVAD days (95% confidence interval [CI] 2.54-4.03) during the first year of LVAD support to 0.78/1000 LVAD days (95%CI 0.37-1.65) during the following third years of LVAD support. Similarly, the incidence of VAD-specific infections during first-year post-LVAD implantation versus during third-year post-LVAD implantation decreased from 0.83/1000 LVAD days (95% CI, 0.53-1.30) to 0.33/1000 LVAD days (95% CI, 0.10-1.04). Figure 1 shows all infections (VAD-specific, VAD-related, and non-VAD) and VAD-specific Infection rates per 1000 days of LVAD support during the first, second, and third years following LVAD implantation. Out of a total of 27 episodes of BSIs, gram-positive organisms were most common in 24 (89%) [*Staph aureus* 9, *Coagulase-negative Staph* 7, *Streptococcus* 4, *Enterococcus* 4]. Table 2 depicts the types of LVAD infections post-LVAD implantation. Most bloodstream (62%) and VAD-specific (56%) infections occurred during the first year post-implantation.

**Table 2:** Infections encountered and characteristics of the total patients (N =89) *SSTI: Skin and soft tissue infection

Total (%)	95
VAD-specific infections	23 (23.5)
Pump/cannula infections	5 (28)
Driveline infections	16 (64)
Surgical site infections	2 (8)
VAD-related infections	16 (16.4)
Bloodstream infections	15 (97)
Mediastinitis	0
Pacer endocarditis	1 (3)
Non-VAD infections	56 (53)
UTI	12 (21)
Pneumonia	16 (29)
SSTI*	8 (14)
Clostridium difficile	3 (5.5)
Other intraabdominal	3 (5.5)
Others	14 (25)

Univariate analysis to evaluate risk factors for increased mortality post-LVAD implantation showed age >60 at LVAD implantation hazard ratio (HR) 1.05 (95% CI 1.01-1.09, p=0.01), presence of infection within 30 days before LVAD implantation HR 2.44 (95% CI 1.09-5.48, p=0.03), underlying ischemic cardiomyopathy HR 2.96 (95% CI 1.28-6.80, p=0.01), and lower ITERMACS profile HR 3.64 (95% CI 1.09-12.13, p=0.04) were found to have an increased risk of death. BSIs post-LVAD implantation did not show a statistically significant increased risk for death HR 1.63 (95% CI 0.56-4.80, p=0.37). Table 3 depicts risk factors for increased mortality post-LVAD implantation with HR and 95% CI.

**Table 3:** Risk factors for mortality post-LVAD implantation *BMI: Body mass index; BTT: Bridge to transplant; CKD: Chronic kidney disease; DT: Destination therapy, BSI: Bloodstream infections, Prior Infections: Infections present within 30 days of LVAD implantation. * INTERMACS definitions as I: Cardiogenic sock, II: Progressive decline on ionotropic support, III: Stable but inotrope dependent, IV: Resting symptoms, V: Exertional intolerance, VI: Exertional limitation, VII: New York Heart Association Class

Parameter	Risk Factors	Hazard Ratio (95% CI)	P-value
Sex	Female	0.56 (0.21, 1.49)	0.24
	Male		
Age at LVAD (>60)	Yes	1.05 (1.01, 1.09)	0.01
	No		
Race	Caucasian		
	Other	0.50 (0.12, 2.12)	0.35
Prior infection	Yes	2.44 (1.09, 5.48)	0.03
	No		
LVAD strategy	DT	3.62 (0.85, 15.47)	0.08
	BTT		
Cardiomyopathy	ICM	2.96 (1.28, 6.80)	0.01
	NICM		
INTERMACS profile	1 to 4	3.64 (1.09, 12.13)	0.04
	5 +		
Diabetes	Yes	0.92 (0.41, 2.06)	0.84
	No		
BMI	BMI>	0.97 (0.92, 1.02)	0.24
Urgency	Emergent	2.35 (1.08, 5.12)	0.03
	Elective		
BSI*	Yes	1.63 (0.56, 4.80)	0.37
	No		

The survival analysis for the combined poor outcome showed a quadratic effect of age, indicating that while a one-year increase in the age at implantation was associated with an increased risk of having a poor outcome, this association was more pronounced at older ages. In addition, underlying ischemic cardiomyopathy, the need for emergent LVAD placement, and BSIs were associated with an increased hazard ratio (HR). BSIs showed an HR of 2.39 (95% CI 1.02-5.57, p=0.04) for a combined poor outcome (Table 4).

**Table 4:** Risk factors for complications in LVAD patients (death, LVAD thrombosis, and stroke) *BMI: Body mass index; BTT: Bridge to transplant; CKD: Chronic kidney disease; DT: Destination therapy, BSI: Bloodstream Infections, Prior Infections: Infections present within 30 days of LVAD implantation. * INTERMACS definitions as I: Cardiogenic sock, II: Progressive decline on ionotropic support, III: Stable but inotrope dependent, IV: Resting symptoms, V: Exertional intolerance, VI: Exertional limitation, VII: New York Heart Association class

Parameter	Risk Factors	Hazard Ratio (95% CI)	P-value
Sex	Female	0.49 (0.21, 1.12)	0.09
	Male		
Age at LVAD	Quadratic at Age 40	0.99 (0.96, 1.02)	
	Quadratic at Age 55	1.04 (1.01, 1.07)	
	Quadratic at Age 70	1.09 (1.02, 1.17)	0.03
Race	Caucasian		
	Other	0.49 (0.15, 1.60)	0.24
Prior Infection	Yes	1.77 (0.86, 3.66)	0.12
	No		
LVAD strategy	DT	2.29 (0.89, 5.90)	0.09
	BTT		
Cardiomyopathy	ICM	2.09 (1.09, 4.02)	0.03
	NICM		
INTERMACS profile	1 to 4	2.35 (1.03, 5.36)	0.04
	5+		
Diabetes	Yes	0.86 (0.44, 1.71)	0.67
	No		
BMI	BMI >	0.99 (0.94, 1.03)	0.51
Urgency	Emergent	2.12 (1.12, 4.01)	0.02
	Elective		
BSI	Yes	2.39 (1.02, 5.57)	0.04
	No		

The most common causes of death were multi-organ failure leading to the withdrawal of support nine (34.6%), acute stroke four (15.3%) (Ischemic-2, hemorrhagic-2), bleeding (non-intracranial), two (7.7%), LVAD-infections two (7.7%), and other causes six (23%). The cumulative all-cause mortality at one, two, and three years was 21%, 28%, and 31%, respectively. 

## Discussion

We analyzed infections post-LVAD implantation at our institution. Earlier studies have suggested LVAD-mediated immune dysfunction leads to an increased risk for infection post-LVAD implantation [8-10]. LVAD type and design have significantly improved over the last decade, and a significant improvement in post-LVAD survival is noted. The effect of prolonged survival on LVAD support on the risk of the development of infections is not known.

We calculated the incidence rate of all infections (VAD-specific+ VAD-related+ non-VAD) and VAD-specific infections only with a prolonged duration of LVAD support. We noticed a significant decrease in all infections and VAD-specific infections with long-term LVAD support. Our findings are very significant; LVAD-mediated immune dysfunction leading to increased infections, as considered earlier, may not be true for new CF LVAD. In our study, the majority of infections were seen within the first six months of LVAD implantation, indicating significant perioperative infections. With prolonged LVAD support, infection rates significantly decreased. As our findings indicate, LVAD may not increase the risk of infections over time, i.e., it may not be immune-compromising. An increased risk for cytomegalovirus (CMV) reactivation post-LVAD implantation has been reported [11].

In our study, one episode of VZV reactivation leading to disseminated varicella infection was noted within two weeks of LVAD implantation. Furthermore, we did not see any other opportunistic infections in our cohort. We believe that post-LVAD CMV reactivation, seen in earlier studies, is a surrogate marker for critical illness in this patient population and may not be associated with LVAD-mediated immune suppression [12-16]. Improvement of heart failure with LVAD support may have contributed to improvement in immune function, which could explain the decreased incidence rate of infections with prolonged LVAD support. A larger study with the control group (patients with end-stage heart failure without LVAD) and the evaluation of cellular and humoral immunity is required to definitively answer this question. The biggest challenge for such a study would be to confound the risk of major surgery in the LVAD group. Our findings are very significant; the earlier thought that LVAD caused immune suppression leading to an increased risk of infection, may not be true.

We also noted that BSIs increased the risk for combined poor outcomes (death+ LVAD thrombosis+ stroke). Most recent INTERMACS data showed infections were the cause of death in only 8% of cases but also mentioned the possibility of an underestimation of infections in this patient population. Our findings that BSIs lead to poor outcomes in terms of thrombosis and stroke are very significant in this respect. A larger study to analyze these variables is needed; we performed a combined analysis as we did not have enough events in individual group.

## Conclusions

The incidence rate of post-LVAD infections decreased significantly over time. LVAD implantation may not be contributing to immune suppression as previously suggested. In our study, BSIs were found to have a significantly increased hazard ratio for a poor outcome post-LVAD implantation.
